# Acupuncture and Related Therapies for Obesity: A Network Meta-Analysis

**DOI:** 10.1155/2018/9569685

**Published:** 2018-09-30

**Authors:** Yanji Zhang, Jia Li, Guoyan Mo, Jing Liu, Huisheng Yang, Xianglin Chen, Hui Liu, Teng Cai, Xian Zhang, Xiangmin Tian, Zhongyu Zhou, Wei Huang

**Affiliations:** ^1^College of Acupuncture and Orthopedics, Hubei University of Chinese Medicine/Hubei Provincial Collaborative Innovation Center of Preventive Treatment by Acupuncture and Moxibustion, Wuhan, China; ^2^Department of Acupuncture, Hubei Provincial Hospital of Traditional Chinese Medicine, Wuhan, China; ^3^Hubei Province Academy of Traditional Chinese Medicine, Wuhan, China; ^4^China Key Laboratory of TCM Resource and Prescription, Hubei University of Chinese Medicine, Ministry of Education, Wuhan, China; ^5^Institute of Acupuncture and Moxibustion of China Academy of Chinese Medical Sciences, Beijing, China

## Abstract

Obesity is a worldwide public health problem. Currently, increasing evidence suggests acupuncture and related therapies are effective for obesity. This network meta-analysis (NMA) was performed to compare the effectiveness of different acupuncture and related therapies. We searched potential randomized controlled trials (RCTs) in three international databases. Thirty-four trials involving 2283 participants were included. Pairwise meta-analysis showed that acupuncture and related therapies were superior to lifestyle modification and placebo in reducing weight and body mass index (BMI). Based on decreases in body weight, results from NMA showed that acupoint catgut embedding (standard mean difference [SMD]: 1.26; 95% credible interval [95% CI], 0.46–2.06), acupuncture (SMD: 2.72; 95% CrI, 0.06–5.29), and combination of acupuncture and related theories (SMD: 3.65; 95% CrI, 0.96–6.94) were more effective than placebo. Another NMA result indicated that acupoint catgut embedding (SMD: 0.63; 95% CI, 0.25–1.11), acupuncture (SMD: 1.28; 95% CrI, 0.43–2.06), combination of acupuncture and related therapies (SMD: 1.44; 95% CrI, 0.64–2.38), and electroacupuncture (SMD: 0.60; 95% CrI, 0.03–1.22) were superior to lifestyle modification in decreasing BMI. Combination of acupuncture and related therapies was ranked the optimal method for both reducing weight and BMI. Further studies will clarify which combination of acupuncture and related therapies is better.

## 1. Introduction

Obesity, a worldwide public health problem, is described as an adiposity-based chronic disease [1]. Currently, guidelines recommended using body mass index (BMI) to classify individuals as having obesity (BMI *⩾*30 kg/m^2^) [2]. Based on the survey conducted previously, the standardized prevalence rates for obesity in adult were 34.9% in United States and 17.7% in China [3, 4]. Moreover, it is associated with other health concerns, such as insulin resistance, type 2 diabetes mellitus, cardiovascular disease, and cancer, which increased individuals and societies' medical burden [5].

Lifestyle modification, pharmacotherapy, and bariatric surgery are considered the mainstay of therapy for obesity [2]. Although diet and exercise play an essential role in the weight management, their precise mode of action remains controversial [6]. Five long-term medicines (naltrexone-bupropion, phentermine-topiramate orlistat, lorcaserin, and liraglutide) have been approved by US Food and Drug Administration (FDA) for the treatment of obesity [7]. The latest research suggested that phentermine-topiramate was associated with the highest possibility of achieving at least 5% weight loss [7]. However, little is known about the long-term safety profile of pharmacotherapy for weight loss. The effectiveness of bariatric procedures for treating obesity has been reported in several randomized controlled trials (RCTs) [8–10]. Nevertheless, the evidence on cardiovascular disease and mortality remains to be validated [11]. Therefore, it is necessary to explore other forms of alternative therapies which are both safe and effective in preventing gaining weight.

In reviewing the literature, it became evident that acupuncture and related therapies have been wildly used for obesity treatment. As mentioned in the meta-analysis, combination of acupuncture and lifestyle modification is more effective compared with lifestyle modification alone [12]. Results of Yeh's research suggested that ear acupoint stimulation had remarkable improvements in the anthropometric parameters of Body Weight (BW), BMI, and so on [13]. In addition, another systematic review performed in 2015 has also shown that clinical efficacy of acupoint catgut embedding therapy was better than that of the control group for simple obesity [14]. However, a major problem is that whether acupuncture or acupuncture-related therapies alone or combined therapy is more effective than lifestyle modification management remains disputable.

By using the technique of network meta-analysis (NMA), both direct and indirect randomized data can be analyzed, and recommended rankings of different treatments can be provided [15, 16]. Therefore, we conducted this Bayesian network meta-analysis to analyse both direct and indirect comparisons of acupuncture and related methods for treating obesity. In this paper, changes in BW, BMI, and the rates of complications of included studies were analyzed.

## 2. Methods

Our research was conducted following the Preferred Reporting Items for Systematic Reviews and Meta-Analyses for Network Meta-Analysis (PRISMA-NMA) checklist [17] (see Appendix 1).

### 2.1. Data Sources and Search Strategy

Three electronic international databases (PubMed/Medline, Embase, and the Cochrane Library) were searched for potential RCTs (randomized controlled trials). We identified articles published from initiation to December 2017 with a limit to studies of RCT and without limitations on language or the form they are published in. The complete search strategies are shown in Appendix 2.

### 2.2. Study Selection

Two researchers (XC and HL) independently identified irrelevant research based on titles and abstracts. Additionally, full-text articles were scanned by these two researchers to identify eligible studies. All disagreements were resolved by consensus and adjudged by a third reviewer (TC) if necessary. In case of duplicate citations, the most updated studies were selected for data extraction.

### 2.3. Inclusion and Exclusion Criteria

The studies included in the NMA met the following criteria: (1) the study design must be a randomized controlled clinical trial (RCT); (2) patients diagnosed with simple obesity irrespective of ages and sex as study subjects; diagnostic criteria must be clear and inclusion and exclusion criteria were explicit; (3) at least one of the following efficacy outcomes or safety endpoints was included: BW, BMI, and adverse events; (4) participants in the experimental group have received acupuncture and related treatments (specifically, classical body acupuncture; electroacupuncture auricular acupoint stimulation; acupoint catgut embedding and warming acupuncture) alone or in combination; (5) English or Chinese language studies.

The following were excluded: (1) self-control and non-RCTs; (2) preclinical studies, systematic reviews, case reports, and meta-analyses; (3) reports without sufficient and clear original data; (4) participants having received other forms of acupuncture such as transcutaneous electrical nerve stimulation or laser acupuncture; (5) duplicate studies and studies reporting the same results.

### 2.4. Data Collection and Quality Assessment

According to a standard data collection sheet, two investigators (TC and XZ) independently extracted the following data: (1) main characteristics of included randomized controlled trials (i.e., year of publication, type of intervention, patients characteristics, types of outcome, and reported adverse events); (2) details of acupuncture and related interventions (i.e., frequency and duration of acupuncture sessions, names of acupuncture points used, and retention time); (3) clinical outcome (i.e., summaries of mean, standard difference, and sample size between treatment groups). In some trials, the change between baseline and after treatment was failed to present. Using the methods recommended in the Cochrane Handbook for Systematic Reviews of Interventions (version 5.1) [18], the missing data was estimated using the following formula:

(1) X−change=X−post−treatment−X−baseline

(2) SDchange=SDbaseline2+SDpost−treatment2−2×r×SDbaseline×SDpost−treatment

where r is a correlation coefficient with a value of 0.5 [19]. For each included RCT, two researches (XT and XC) independently assessed their risk of bias by the Cochrane Collaboration tool [20]. Bias risks of each study were assessed from six aspects: random sequence generation, allocation concealment, blinding of participants and investigators, blinding of outcome assessment, incomplete outcome data addressed, and selective outcome reporting, while ranked in high risk, low risk, and unclear risk.

### 2.5. Statistical Analysis

Firstly, standard pairwise meta-analysis was initially performed using the Review Manager (Version 5.3, Cochrane Collaboration, Oxford, UK). We calculated I-square (I^2^) test to assess heterogeneity among RCTs [21]. To be specific, when there was I^2^ > 50%, they were analysed using a random effects model; otherwise, a fixed effect model was chosen. Subgroup analyses were conducted according to the type of acupuncture treatment and the treatment of control group. Mean difference (MD) with 95% confidence intervals (CI) was used to analyze continuous data. We generated forest plots to illustrate the relative strength of curative effects.

Second, to indirectly compare the effectiveness among treatments of acupuncture and related therapies, we did a random effects model NMA within a Bayesian framework, by using WinBUGS (Version 1.4.3, MRC Biostatistics Unit, Cambridge, UK) [22, 23]. Models were computed with Markov chain Monte Carlo (MCMC) simulation methods, using four chains with overdispersed initial values. We utilized the Markov chains for 50,000 simultaneous iterations after the first 20000 iterations were discarded because they may have an influence on the arbitrary value. In this process, the convergence of the model was assessed by the Brooks-Gelman-Rubin (BGR) method; a value of potential scale reduction factor (PSRF) close to 1 indicated the better convergence [24]. The continuous outcome was measured by a standard mean difference (SMD) with a 95% credible intervals (CrI) for indirect comparisons.

Finally, plot of surface under the cumulative ranking curve (SUCRA) was generated using the STATA software (Version 13.0; Stata Corporation, College Station, Texas, USA), which indicated the probability of each intervention of being ranked best [25]. In our study, higher SUCRA scores mean the higher rank of the treatment [15]. A Z value and its corresponding p-value were calculated, and an R value less than 0.05 indicated a statistically significant difference.

## 3. Results

### 3.1. Study Search

**Figure 1:**
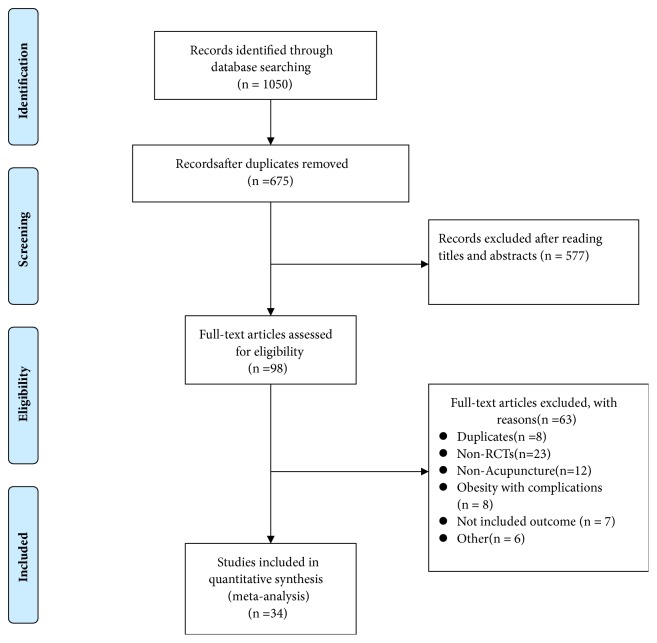
PRISMA flow chart.

We performed this research on Dec 26 2017. As shown in Figure 1, a total of 1050 records were initially identified from the databases. 675 studies left after duplicates were removed. 577 records were excluded after carefully scanning titles and abstracts. Finally, 34 trials with 2283 participants were included in our NMA [26–59], covering 8 groups, manual acupuncture; electroacupuncture; auricular acupoint stimulation; acupoint catgut embedding; pharmacotherapy; warming acupuncture; lifestyle modification; placebo.

### 3.2. Study Description

**Figure 2:**
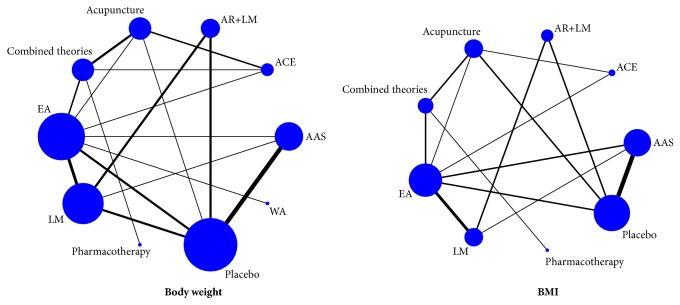
*Network plot.* BMI: body mass index; LM: lifestyle modification; AAS: auricular acupoint stimulation; EA: electroacupuncture; ACE: acupoint catgut embedding; WA: warming acupuncture; AR: acupuncture and related therapies; combined therapies: combination of acupuncture and related therapies.

Main characteristics of included RCTs were shown in Table 1. The participants were from Australia [28], the United States [26], Turkey [46], Korea [51], Iran [36], Egypt [48], and China. Age of participants ranged from 15 to 70 years, while the sample size of the studies ranged from 12 to 86. Among the included RCTs, there were one four-arm trials, 5 three-arm trials, and 28 two-arm trials. Fourteen studies compared acupuncture to placebo. Ten studies compared acupuncture to lifestyle intervention. Six studies compared combined therapies to acupuncture alone. Details about acupuncture points used, retention time, frequency, and duration of acupuncture sessions were shown in Table 2. In these research, 30 articles [26–30, 32, 34–38, 41–59] reported the weight loss, while 25 articles reported the change in BMI. The details of mean, standard difference (SD), and sample size between different groups for eligible studies are summarized in Appendix 3. The Cochrane risk of bias assessment was presented in Table 3. Furthermore, the network plot of included comparisons was shown in Figure 2.

### 3.3. Pairwise Meta-Analyses

#### 3.3.1. Body Weight

A direct pairwise meta-analysis showed that acupuncture and related therapies showed a greater BW reduction than lifestyle modification (MD: 1.66; 95% Confidence interval, 0.63to2.70) and placebo (MD: 1.15; 95% CI, 0.67to1.63). When compared to acupuncture, combination of acupuncture and related theories showed a marginally stronger effect in weight loss (MD: 1.56; 95% CI, 0.07to3.05). There was no statistically significant difference between combination of acupuncture and related theories and pharmacotherapy in their effectiveness in BW (MD: 2.44; 95% CI, -1.98to6.86). (Table 4)

*BMI*. As for the comparison in reducing BMI, acupuncture and related therapies were found to be marginally superior to lifestyle modification (MD: 1.17; 95% CI, 0.09to2.26) and placebo (MD: 0.57; 95% CI, 0.40to0.74). The remaining direct comparisons did not show significant differences (Table 4).

### 3.4. Network Meta-Analysis

#### 3.4.1. Body Weight

The NMA showed that all treatments other than acupuncture combined lifestyle modification were more efficacious than lifestyle modification. Three treatments were significantly more effective than placebo. Specifically, acupoint catgut embedding (SMD: 1.26; 95% credible interval, 0.46to2.06), acupuncture (SMD: 2.72; 95% CrI, 0.06to5.29), and combination of acupuncture and related therapies (SMD: 3.65; 95% CrI, 0.96to6.94). Furthermore, moxibustion with warming needle was associated with a significantly improvement than lifestyle modification (SMD: -5.24; 95% CrI, -10.15to-0.55) (Table 5).

#### 3.4.2. BMI

Four treatments showed superiority over placebo, including acupoint catgut embedding (SMD: 1.31; 95% CrI, 0.36to2.06), acupuncture (SMD: 1.94; 95% CrI, 0.83to3.00), combination of acupuncture and related theories (SMD: 3.65; 95% CrI, 0.96to6.94), and electroacupuncture (SMD: 1.28; 95% CrI, 0.43to2.11). Four treatments were significantly more effective than lifestyle modification, including acupoint catgut embedding (SMD: 0.63; 95% CI, 0.25to1.11), acupuncture (SMD: 1.28; 95% CrI, 0.43to2.06), combination of acupuncture and related theories (SMD: 1.44; 95% CrI, 0.64to2.38), and electroacupuncture (SMD: 0.60; 95% CrI, 0.03to1.22). Also, the combination of acupuncture and related theories and acupuncture alone were both superior to the acupuncture combined lifestyle modification in their ability to reduce body mass index (SMD = -1.76, 95% CrI =−2.96 to −0.62; SMD = −1.59, 95% CrI = −2.71to −0.34) (Table 5).

### 3.5. Ranking

#### 3.5.1. Body Weight

**Figure 3:**
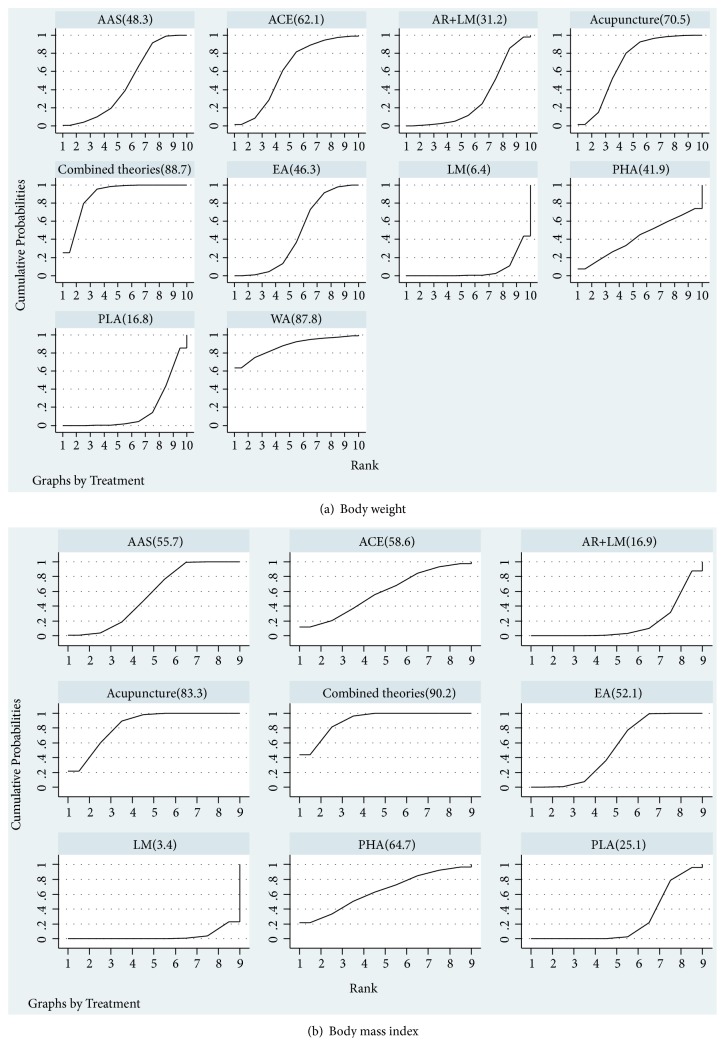
*Surface under the cumulative ranking curves*. LM: lifestyle modification; AAS: auricular acupoint stimulation; EA: electroacupuncture; ACE: acupoint catgut embedding; WA: warming acupuncture; AR: acupuncture and related therapies; combined therapies: combination of acupuncture and related therapies; PLA: placebo; PHA: pharmacotherapy.

Ranking of the different treatment methods was displayed Figure 3. The results suggested that, on the aspect of weight loss, combination of acupuncture and related therapies was ranked the optimal method, the best, (88.7%), followed by moxibustion with warming needle (87.8%), manual acupuncture (70.5%), acupoint catgut embedding (ACE,62.1%), auricular acupoint stimulation (AAS,48.3%), electro acupuncture (EA,46.3%), pharmacotherapy (41.9%), acupuncture combined lifestyle modification (AR+LM,31.2%), placebo acupuncture/sham acupuncture (16.8%), and lifestyle modification (LM,6.4%) which was ranked as the worst.

#### 3.5.2. BMI

The results suggested that, on the aspect of BMI, combination of acupuncture and related therapies was ranked the optimal method, the best, (90.2%), followed by manual acupuncture (83.3%), pharmacotherapy (64.7%), acupoint catgut embedding (58.6%), auricular acupoint stimulation (55.7%), electroacupuncture (52.1%), placebo acupuncture/sham acupuncture (25.1%), acupuncture combined lifestyle modification (16.9%), and lifestyle modification (3.4%) which was ranked as the worst.

### 3.6. Inconsistency Assessment

#### 3.6.1. Body Weight

The Z test illustrates the inconsistency of the NMA specifically (Appendix 4). For the inconsistency test outcome of BW, 95% CI of 8 loops was included 0, which reflected that no significant inconsistency was found. However, another 2 loops (ACE-Acupuncture-Combined therapies; ACE-Combined theories-EA) were found statistical inconsistency between direct and indirect comparisons.

#### 3.6.2. BMI

For the inconsistency test outcome of body mass index, 95% CI of all loops (acupuncture -combined theories-EA; acupuncture-EA-placebo; AR+LM -EA- LM-placebo; ACE-acupuncture-EA; AAS-EA-LM; AAS-AR+LM-LM-placebo; AAS-EA-placebo) were included 0, which reflected that no significant inconsistency was found.

### 3.7. Safety

Ten RCTs [26–28, 31, 32, 35, 36, 38, 39, 45] reported adverse events, while no major complications were noticed in all included studies. Three included studies [35, 36, 39] reported that no adverse effects were noted in both experimental group and placebo group. In one included RCT, there were two patients reporting mild ecchymosis and one abdominal discomfort case reported as adverse events after electroacupuncture treatment; no case was reported in the lifestyle modification group [27]. In another study, there were seven subjects in group auricular acupoint stimulation and two subjects in group placebo had mild tenderness [32].

## 4. Discussion

The aim of this study was to identify the efficacy and safety of acupoint stimulation therapy for obesity. In this NMA, the association of each acupuncture and related therapies with relative weight loss was compared by the combination of direct and indirect evidence from 34 RCTs in 2283 obese patients.

This study has three key findings. First, ranking graphs of the primary outcome suggested that the combination of acupuncture and related therapies was the most effective in losing weight and improving BMI. Second, compared with placebo or sham acupuncture, combination of acupuncture-related therapies, manual acupuncture, acupoint catgut embedding, auricular acupuncture therapy, and electroacupuncture are all associated with higher odds of achieving weight loss. Third, combination of acupuncture and related therapies, manual acupuncture, pharmacotherapy, acupoint catgut embedding, auricular acupoint stimulation, and electroacupuncture were superior to lifestyle intervention.

Lifestyle modification, like diet intervention and physical activity, is recommended as safe and effective way to lose weight [60]. Results of direct and indirect evidence suggest acupuncture and related theories had significant beneficial effects in dealing with obesity compared with lifestyle modification. Both experimental and clinical data prove the efficacy of acupuncture for obesity [61]. Experimental data suggests that acupuncture exerts beneficial effects on weight loss [62, 63]. The majority of clinical evidence suggests that acupuncture and related therapies reduced more weight than sham control group [26, 28, 31, 32], which are consistent with our results. Previous animal studies have observed that the expression of obesity-related peptides was upregulated in the hypothalamus after acupuncture treatment, which induced less food intake and weight loss [62, 64, 65]. Similarly, significant decreases in plasma leptin level were observed after EA treatment in obese patients [46]. With regard to insulin level, several experimental studies reported that EA can improve insulin sensitivity [66, 67]. However, results from clinical trials regarding insulin levels are controversial. Cabioğlu MT reported that EA increased insulin level compared with control group [56], but Gucel F indicated that acupuncture decreased insulin level [46]. As to effects on lipid metabolism, acupuncture was reported to be effective in decreasing total cholesterol (TC), triglycerides (TG) and LDL-C concentrations [68, 69] of obese rat. Significant decreases in TC [55], TG [35], and LDL-C [55] were observed whereas no changes in HDL-C [55] levels were observed in clinical trials. Furthermore, experimental studies suggest that there was significant decrease in serum TNF*α* after EA [70]. Except for the noted mechanisms, EA can also induce white adipose tissue (WAT) browning via increasing uncoupling protein-1 (UCP1) gene expression [71].

This NMA has several attractive advantages. We focused on simple obesity patients without complication, which decreased the heterogeneity and improve the quality of this study. In addition, we compared acupuncture and acupuncture-related therapies with the first-line treatment for obesity-lifestyle modification with a Bayesian framework. The rank test of effectiveness provides data to favour acupuncture and acupuncture-related therapies. Lastly, we conducted a comprehensive search and included all eligible studies. We compared five different acupuncture treatments (manual acupuncture; electro acupuncture; auricular acupoint stimulation; acupoint catgut embedding; moxibustion with warming needle) in the clinical effectiveness in treating patients with obesity.

However, this study has several limitations. First, we failed to evaluate the safety of each acupoint stimulation therapy due to the limited data in primary studies. Future trials should report adverse events clearly to improve the quality of study design. Second, unaddressed concerns still exist regarding the long-term effects of using acupuncture and acupuncture-related therapies on weight management in a clinical setting. The duration of acupuncture sessions and follow-up duration of most included trials ranging from four weeks to twelve weeks. Further clinical evaluation of acupuncture for obesity with longer follow-up appears warranted. Third, blinding of patients and researches was not applied among included studies and the included trials were mainly conducted in China, which may lead to publication bias [72]. Fourth, included study in our NMA lack of research compares the effectiveness between acupuncture, pharmacotherapy, and different types of combination of acupuncture. Further confirmatory comparative effectiveness trials should compare different types of combination of acupuncture. Except one study compared acupuncture and pharmacotherapy [29], additional research is needed to further explore. Finally, we use R-value to estimate the changes in standard deviations (SD), which might enlarge the SD compared with the originals values.

Overall, our results indicate that combination of acupuncture and related therapies ranks as the optimal method for reducing both weight and BMI. Further studies will clarify which combination of acupuncture and related therapies is better.

## Figures and Tables

**Table 1:** Main characteristics of included randomized controlled trials.

Study ID and Country	Sample size R/A	Age: mean (SD) or range R/A	Intervention	Control	Adverse events reported R/A	Type of outcomes
Allison et al. [17] 1995, USA	35/34	19 - 70	AAS	Placebo	Redness, pain, bleeding	BW,

Hsu et al. [18] 2005, Taiwan	24/22	41.5(11.2)/41.0 (10.0)	EA	LM	Ecchymosis(2), abdominal discomfort(1)/None	BW, BMI

Richards et al. [19] 1998, Australia	28/32	44.1 (11.7)/43.0 (13.6)	AAS	Placebo	intercurrent illness and discontinued(1)/None	BW

He et al. [20] 2008, China	40/40	18 - 50	Combined therapies#	Pharmacotherapy	NR	BW, BMI

Li et al. [21] 2006, China	26/30	16.00(1.38)/16.00(1.95)	EA	LM	NR	BMI
	29/30	15.00(2. 04)/16.00(1.95)	AAS			

Tong et al. [22] 2011, China	76/42	35.08(9.31)/34.60(8. 55)	Acupuncture	Placebo	Adverse events VAS	BMI

Hsu et al. [23] 2009, Taiwan	23/22	40.0 (10.5)/39.4 (13.6)	AAS	Placebo	Minor-inflammation(1), mild tenderness (7)/mild tenderness (2)	BW, BMI

Hsieh et al. [24] 2010, Taiwan	26/26	18 - 20	AAS	Placebo	NR	BMI

Hsieh et al. [25] 2011, Taiwan	27/28	18 - 20	AAS	Placebo	NR	BW

Abdi et al. [26] 2012, Iran	86/83	37.29(1.0)/38.73 (1.1)	AAS	Placebo	None	BW, BMI

Darbandi et al. [27] 2012, Iran	43/43	37.57(9.26)/37.65(9.71)	AR+ LM	Placebo	None	BW, BMI,

He et al. [28] 2012, China	30/30	18-54	AR+ LM	LM	NR	BW, BMI

Lien et al. [29] 2012, Taiwan	24/23	39.2(11.6)/40.7 (9.7)	AAS	Placebo	Dizziness (1)/None	BW, BMI

Darbandi et al. [30] 2014, Iran	20/20	38.0(0.9)/38.0(1.3)	EA	Placebo(EA)	None	BW, BMI
	20/20	39.0(1.8)/37.9(1.5)	AAS	Placebo(AAS)		

Yeh et al. [31] 2015, Taiwan	36/34	29.9 (7.7)/32.8 (9.5)	EA	Placebo	NR	BMI

Chen et al. [32] 2007, China	40/40	43.1(13.6)/44.6(10.3)	ACE	Acupuncture	NR	BW, BMI

Huang et al. [33] 2011, China	30/30	NR	ACE	EA	NR	BW, BMI

Tang et al. [34] 2009, China	33/32	21-54/22-55	Combined therapies	EA	NR	BW, BMI

Shi et al, [35] 2006, China	40/42	17-49/18-51	Combined therapies	EA	NR	BW, BMI

Hsu et al. [36] 2005, Taiwan	22/20	40.0 (11.5)/41.3 (9.9)	EA	LM	mild Ecchymosis(3), abdominal discomfort(1)/None	BW, BMI

Güçel et al. [37] 2012, Turkey	20/20	34.6±6.3/36.8±7.8	Acupuncture	Placebo	NR	BW, BMI

Deng et al. [38] 2014, China	30/30	32(7)/33(7)	Combined therapies	Acupuncture	NR	BW
	30/30	32(7)/33(8)		ACE		

Hassan et al. [39] 2014, Egypt	21/30	45.00 (9.32)/43.47 (9.59)	AR+ LM	LM	NR	BW, BMI

He et al. [40] 2014, China	28/28	NR	Combined therapies	Acupuncture	NR	BW, BMI

Wang et al. [41] 2013, China	45/45	31(10)/32(12)	EA	Acupuncture	NR	BMI

Sujung et al. [42] 2014, South Korea	22/15	34.7(11.9)/42.7(10.2)	AAS	Placebo	NR	BW, BMI

Bu et al. [43] 2007, China	32/23	32.1(1.1)/33.4(1.3)	Combined therapies	Acupuncture	NR	BW, BMI

Shi et al. [44] 2005, China	36/32	19~58/18~56	WA	EA	NR	BW

Yang et al. [45] 2010, China	31/30	18~42/18~48	AR+ LM	LM	NR	BW

Cabioglu et al. [46] 2005, Turkey	22/12	39.8(5.3)/43.3(4.3)	EA	Placebo	NR	BW
	22/21	39.8(5.3)/42.7(3.9)		LM		

Cabioglu et al. [47] 2006, Turkey	20/15	42.1(4.4)/41.8(4.6)	EA	Placebo	NR	BW
	20/15	42.1(4.4)/42.9 (4.3)		LM		

Cabioglu et al. [48] 2008, Turkey	20/15	40.55 (5.30)/41.47 (4.61)	EA	Placebo	NR	BW
	20/23	40.55 (5.30)/42.91(4.02)		LM		

Darbandi et al. [49] 2013, Iran	42/44	36.50 (9.26)/36.48 (8.69)	AR+ LM	Placebo	NR	BW, BMI

Fogarty et al. [50] 2015, Australia	19/16	>18	AR+ LM	Placebo	NR	BMI

BW: body weight; BMI: body mass index; LM: lifestyle modification; AAS: auricular acupoint stimulation; EA: electroacupuncture; ACE: acupoint catgut embedding; WA: warming acupuncture; AR: acupuncture and related therapies; #combination of acupuncture and related therapies.

**Table 2:** Descriptions of the included acupuncture and related therapies.

Study ID (Country)	Style of acupuncture	Names of acupuncture points used	Retention time	Frequency & duration of Acupuncture sessions
Allison et al. 1995, USA	AAS	NR	2-3 min	3 sessions daily for 12 weeks

Hsu et al. 2005, Taiwan	EA	Qiai(REN9), Shuifen(REN9) Shuidao(ST28), Siman(K14) Zusanli(ST26), Fenglong(ST40) Sanginjao(SP6)	40 min	2 sessions weekly for 6 weeks

Richards et al. 1998, Australia	AAS	Shenmen(TF4), Stomach(CO4)	15-20 min	2 sessions daily for 4 weeks

He et al. 2008, China	Combined therapies#	Ear acupressure: Shenmen(TF4), Neifenmi(CO18), Pi(CO13), Wei(CO14), Sanjiao(CO17), Dachang(CO7), Naodian Body acupuncture: Tianshu(ST25), Guanyuan(RN4) Sanyinjiso(SP9), Fenglong(ST40) Zusanli(ST36)	Ear acupressure:3 days Body acupuncture:30 min	Ear acupressure:1 session every 3 days with a total of 10 sessions Body acupuncture: The first 5 days of treatment 1 time, 5 days after treatment 1, 1 month, for a course of treatment.

Li et al. 2006, China	EA	Sanginjao(SP6), Tianshu(ST25) Zusanli(ST36), Quchi(LI11) Fenglong(ST40), Neiting(ST44) Zhongwan(CV12), Pishu(BL20) Shenshu(BL23), Qihai(CV6) Yinlingquan(SP9), Shangjuxu(ST37) Taichong( LR3)	10 min	1 session daily with a total of 60 sessions, 2 days rest in-between 10 sessions
	AAS	Hunger point Pizhixia(AT4) Shenmen(TF4), Shenshangxian(TG2P) Sanjiao(CO17), Pi(CO13) Wei(CO14), Fei(CO14) Kou(CO1), Dachang(CO7) Zhichangxiduan(HX2)	15-20 min	1 session daily with a total of 10 sessions for 10 weeks, 2 day rest in-between 10 sessions

Tong et al. 2011, China	Acupuncture	Zhongwan(CV12), Zhongji(CV3) Daheng(SP15), Xiawan(CV10) Shimen(CV5), Tianshu(ST25) Liangqiu(ST34), Zusanli(ST36) Yinlingquan(SP9)	30 min	1 session every other day for a total of 5 weeks with 12 sessions

Hsu et al. 2009, Taiwan	AAS	Hunger point, Shenmen point(TF4) Stomach point(CO4), Endocrine point(CO18)	3 days	2 sessions weekly for a total of 6 weeks with 12 sessions

Hsieh et al. 2010, Taiwan	AAS	NR	2/3 days	1 session weekly for 10 sessions

Hsieh et al. 2011, Taiwan	AAS	NR	NR	1 session weekly for a total of eight weeks

Abdi et al. 2012, Iran	AAS	Shenmen(TF4), Stomach(CO4) Hunger point Mouth(CO1) Centre of ear(HX1), Sanjiao(CO17)	3 days	Twice a week for a total of 6 weeks

Darbandi et al. 2012, Iran	AAS	Shenmen(TF4), Stomach(CO4) Hunger point Mouth(CO1) Centreof ear(HX1), Sanjiao(CO17)	3 days	Twice a week for a total of 6 weeks

He et al. 2012, China	AAS	Hunger point Stomach(CO4) Spleen(CO14), LargeIntestine(CO7) Endocrine(CO18), Shenmen(TF4)	3 days	3 times a day for 4 weeks

Lien et al. 2012, Taiwan	AAS	Shenmen point(TF4), Stomach point(CO4) Hunger point, Endocrine point(CO18)	NR	3 session weekly with a total of 12 sessions for 4 weeks
	Placebo	Shenmen point(TF4), Stomach point(CO4) Hunger point, Endocrine point(CO18)	NR	3 sessions weekly with a total of 12 sessions for 4 weeks

Darbandi et al. 2014, Iran	EA	Tianshu (ST-25), Weidao(GB28) Zhongwan(REN12), Shuifen(REN9) Guanyuan(REN4), Sanyinjiao(SP6) Quchi(LI11), Fenlong(ST40) Qihai(REN6), Yinlingquan (SP9)	20 min	2 sessions weekly for a total of 6 weeks
	AAS	Shenmen (TF4), Stomach (CO4) Hunger point, Mouth (CO1) Center of ear (HX1), Sanjiao (CO17)	3 days	2 sessions weekly for a total of 6 weeks

Yeh et al. 2015, Taiwan	EA	Shenmen (TF4), Stomach CO4) Endocrine (CO18) Hunger point	20 min	NR

Chen et al. 2007, China	ACE	Liangqiu(ST34), Zhongwan(CV12) Tianshu(ST25), Shuifen(CV9) Fenglong(ST40)	A week	1 session weekly with a total of 30 sessions for 4 weeks
	Acupuncture	Liangqiu(ST34), Zhongwan(CV12) Tianshu(ST25), Shuifen(CV9) Fenglong(ST40)	45 min	The first 5 days are 1 times a day, and 1 time after 5 days, 1 month is 1 course of treatment.

Huang et al. 2011, China	ACE	One set is Tianshu(ST25) Zhongwan(CV12), Guanyuan(CV4) Zusanli(ST36), Weishu(BL21) Ashixue	24 hour	1 session weekly with a total of 7 sessions for 60 days
	EA	Zhongwan(CV12), Tianshu(ST25) Daheng(SP15), Shuifen(CV9) QIhai(CV6), Guanyuan(CV4) Zusanli(ST36), Ashixue	30 min	3 sessions weekly with a total of 12 sessions for 60 days

Tang et al. 2009, China	Combined therapies	EA: Zhongwan(CV12), Xiawan(CV10) Guanyuan(CV4), Tianshu(ST25) Shuifen(CV9), Sanyinjiao(SP6) Zusanli(ST36), Xuehai(SP10) Xinshu(BL15), Geshu(BL17) Pishu(BL20) ACE: Zhongwan(CV12), Tianshu(ST25) Qihai(CV6), Tianshu(ST25) Liangqiu(ST34), Zusanli(ST36) Gongsun(SP4), Xinshu(BL15) Pishu(BL20)	EA:30 min	EA:The first 3 days are 1 times a day, and 1 time after 3 days, 15 days is 1 course of treatment. ACE:After the first acupoint catgut embedding for 3 consecutive times, the interval is buried for the second time after 15 days, and the acupuncture is performed for the third time after the end of the treatment period.
	EA	Zhongwan(CV12), Xiawan(CV10) Guanyuan(CV4), Tianshu(ST25) Shuifen(CV9), Sanyinjiao(SP6) Zusanli(ST36), Xuehai(SP10) Xinshu(BL15), Geshu(BL17) Pishu(BL20)	30 min	The first 3 days are 1 times a day, and 1 time after 3 days, 15 days is 1 course of treatment.

Shi et al. 2006, China	Combined therapies	Zhongwan(CV12), Xiawan(CV10) Qihai(CV6), Zhongji(CV3) Tianshu(ST25), Daheng(SP15) Liangmen(ST21), Huaroumen(ST24) Shuidao(ST28), Quchi(CV6) Zhigou(TE6), Hegu(LI4) Liangqiu(ST34), Zusanlli(ST36) Shangjuxu(ST37), Fenglong(ST40) Sanyinjiao(SP6), Gongsun(SP4) Neiting((ST44)	30 min	EA:The first 3 days are 1 times a day, and 1 time after 3 days, 15 days is 1 course of treatment. ACE: After the first acupoint catgut embedding for 3 consecutive times, the interval is buried for the second time after 15 days, and the acupuncture is performed for the third time after the end of the treatment period.
	EA	Zhongwan(CV12), Xiawan(CV10) Qihai(CV6), Zhongji(CV3) Tianshu(ST25), Daheng(SP15) Liangmen(ST21), Huaroumen(ST24) Shuidao(ST28), Quchi(CV6) Zhigou(TE6), Hegu(LI4) Liangqiu(ST34), Zusanlli(ST36) Shangjuxu(ST37), Fenglong(ST40) Sanyinjiao(SP6), Gongsun(SP4) Neiting((ST44)	30 min	The first 3 days are 1 times a day, and 1 time after 3 days, 15 days is 1 course of treatment.

Hsu et al. 2005, Taiwan	EA	Qihai (REN-6), Shuifen (REN-9) Shuidao (ST-28), Siman (K-14) Zusanli (ST-26), Fenglong(ST-40) Sanginjao (SP-6)	40 min	2 sessions weekly with a total of 12 sessions for 6 weeks

Güçel et al. 2012, Turkey	Acupuncture	Hegu(LI4), Shenmen(HT7) Zusanli(ST36), Neiting(ST44) Sanyinjiao(SP6)	20 min	2 sessions weekly with a total of 10 sessions for 5 weeks

Deng et al. 2014, China	Combined therapies	Zhongwan (CV 12), Xiawan(CV 10) Qihai(CV 6), Guanyuan( CV4) Huaroumen (ST 24), Wailing ( ST 26) Daheng (SP 15), Tianshu (ST 25) Yinjiao (CV 7), Zhigou (TE 6) Zusanll (ST 36)	NR	Acupuncture:1 session every 3 days with a total of 21 sessions for 4 weeks, 3 days rest between every session Acupoint catgut Embedding:1 session weekly with a total of 3 sessions for 3 weeks
	Acupuncture	Zhongwan (CV 12), Xiawan(CV 10) Qihai(CV 6), Guanyuan( CV4) Huaroumen (ST 24), Wailing ( ST 26) Daheng (SP 15), Tianshu (ST 25)	30 min	1 session every 3 days with a total of 21 sessions for 4 weeks, 3 days rest between every session
	Acupoint catgut embedding	Zhongwan (CV 12), Tianshu (ST 25) Yinjiao (CV 7), Zhigou (TE 6) Guanyuan( CV4), Zusanli (ST 36)	NR	1 session weekly with a total of 3 sessions for 3 weeks

Hassan et al. 2014, Egypt	AR	NR	NR	NR

He et al. 2014, China	AR	NR	NR	NR
	acupuncture	Tianshu (ST25), Liangmen(ST21) Daheng (SP15), Zusanli (ST36) Sanyinjiao(SP6), Quchi (LI11) Zhigou (SJ6), Zhongwan(RN12) Qihai (RN06)	30 min	1 session daily with a total of 21 sessions for 3 weeks

Wang et al. 2013, China	EA	Neiting(ST44), Shangjuxu(ST37) Xiajuxu(ST39), Fenglong(ST40) Tianshu(ST25), Zusanli(ST36) Quchi(LI11)	30 min	1 session every 2 days with a total of 12 sessions for 3 weeks
	Acupuncture	Neiting(ST44), Shangjuxu(ST37) Xiajuxu(ST39), Fenglong(ST40) Tianshu(ST25), Zusanli(ST36) Quchi(LI11)	30 min	1 session every 2 days with a total of 12 sessions for 3 weeks

Sujung et al. 2014, South Korea	AAS	Shen-men(TF4), Stomach(CO4) Spleen(CO13), Hunger point Endocrine(C018)	NR	1 session weekly with a total of 8 sessions for 8 weeks

Bu et al. 2007, China	Combined therapies	Acupuncture: Tianshu(ST25), Guanyuan(CV4) Zusanli(ST36), Fenglong(ST40) Sanyinjiao(SP6) AAS: Shenmen(TF4), Endocrine(C018) Spleen(CO13), Stomach(CO4) Dachang(CO7), Sanjiao(CO17 ) Naodian	Acupuncture:30 min ear acupressure:1 day	1 session every day with a total of 10 sessions for 6 weeks, 1 week rest in-between 10 sessions.
	Acupuncture	Tianshu(ST25), Guanyuan(CV4) Zusanli(ST36), Fenglong(ST40) Sanyinjiao(SP6)	30 min	1 session every day with a total of 10 sessions for 6 weeks, 1 week rest in-between 10 sessions.

Shi et al. 2005, China	Warming acupuncture	Zhongwan(CV12), Shuifen(CV9) Qihai(CV6), Zhongji(CV3) Tianshu(ST25), Shuidao(ST28) Neiguan(PC6), Hegu(LI4) Xuehai(SP10), Zusanli(ST36) Fenglong(ST40), Sanyinjiao(SP6)	40 min	1 session every day with a total of 15 sessions for 4 weeks
	EA	Zhongwan(CV12), Shuifen(CV9) Qihai(CV6), Zhongji(CV3) Tianshu(ST25), Shuidao(ST28) Neiguan(PC6), Hegu(LI4) Xuehai(SP10), Zusanli(ST36) Fenglong(ST40), Sanyinjiao(SP6)	40 min	1 session every day with a total of 15 sessions for 4 weeks

Yang et al. 2010, China	AR	Zhongwan(CV12), Tianshu(ST25) Guanyuan(CV4), Zusanli(ST36) Fenglong(ST40), Yinlingquan(SP9) Sanyinjiao(SP6), Pishu(BL20) Weishu(BL21), Ashixue	30 min	1 session daily with a total of 15 sessions for 7 weeks, 3 days rest between every session

Cabioglu et al. 2005, Turkey	EA	Body points: Hegu(LI 4), Tianshu(ST 25) Quchi(LI 11), Zusanli(ST 36) Neiting(ST 44	30 min	Body EA was performed everyday, and EA was performed every other day

Cabioglu et al. 2006, Turkey	EA	Body points: Quchi(LI 11), Zusanli(ST 36) Neiting(ST 44)	30 min	Body EA application was performed daily for 20 days, and EA was applied to each ear on alternating days

Cabioglu et al. 2008, Turkey	EA	Body points: Hegu(LI 4), Quchi(LI 11) Tianshu(ST 25)Zusanli(ST 36) Taitong(Liv 3), Neiting(ST 44)	30 min	Body EA application was performed daily for 20 days, and EA was applied to each ear on alternating days

Darbandi et al. 2013, Iran	AR	Inrervention group: Tianshu(ST 25), Weidao(GB 28) Zhongwan(RN 12), Shuifen(RN 9) Guanyuan(RN 4), Sanyinjiao(SP 6) Excess group: Quchi(LI 11), Fenglong(ST 40) Deficiency group: Qihai(RN6), Yinlingquan(SP9)	20 min	Two treatmennt per week for a total of 6 weeks(12 treatments)

Fogarty et al. 2015, Australia	AR	Hegu(LI 4), Quchi(LI 11) Zusanli(ST 36), Neiting(ST 44) Taichong(LR 3) Auricular acupuncture: Shenmen(TF4)	NR	NR

LM: lifestyle modification; AAS: auricular acupoint stimulation; EA: electroacupuncture; ACE: acupoint catgut embedding; WA: warming acupuncture; AR: acupuncture and related therapies; #combination of acupuncture and related therapies.

**Table 3:** Risk of bias assessment.

Study	Random sequence generation	Allocation concealment	Blinding of participants and investigators	Blinding of outcome assessment	Incomplete outcome data addressed	Selective outcome reporting
Allison et al. 1995, USA	Unclear risk	Unclear risk	High risk	Low risk	Low risk	Unclear risk
Hsu et al. 2005, Taiwan	Unclear risk	Unclear risk	High risk	Low risk	Low risk	Unclear risk
Richards et al. 1998, Australia	Unclear risk	Low risk	High risk	Low risk	Low risk	Unclear risk
He et al. 2008, China	Unclear risk	Unclear risk	High risk	Low risk	Low risk	Unclear risk
Li et al. 2006, China	Low risk	Unclear risk	High risk	Low risk	Low risk	Unclear risk
Tong et al. 2011, China	Unclear risk	Unclear risk	High risk	Low risk	Low risk	Unclear risk
Hsu et al. 2009, Taiwan	Unclear risk	Unclear risk	High risk	Low risk	Low risk	Unclear risk
Hsieh et al. 2010, Taiwan	Unclear risk	Unclear risk	High risk	Low risk	High risk	Unclear risk
Hsieh et al. 2011, Taiwan	Unclear risk	Unclear risk	High risk	Low risk	Low risk	Unclear risk
Abdi et al 2012, Iran	Unclear risk	Unclear risk	High risk	Low risk	High risk	Unclear risk
Darbandi et al 2012, Iran	Unclear risk	Unclear risk	High risk	Low risk	Low risk	Unclear risk
He et al 2012, China	Unclear risk	Unclear risk	High risk	Low risk	Unclear risk	Unclear risk
Lien et al 2012, Taiwan	Low risk	Low risk	High risk	Low risk	High risk	Unclear risk
Darbandi et al 2014, Iran	Low risk	Low risk	High risk	Low risk	Low risk	Unclear risk
Yeh et al. 2015, Taiwan	Low risk	Low risk	High risk	Low risk	High risk	Unclear risk
Chen et al. 2007, China	Low risk	Unclear risk	High risk	Low risk	Low risk	Unclear risk
Huang et al. 2011, China	Unclear risk	Unclear risk	High risk	Low risk	Low risk	Unclear risk
Tang et al. 2009, China	High risk	Unclear risk	High risk	Low risk	Low risk	Unclear risk
Shi et al, 2006, China	Low risk	Unclear risk	High risk	Low risk	Unclear risk	Unclear risk
Hsu et al. 2005, Taiwan	Low risk	Unclear risk	High risk	Low risk	Low risk	Unclear risk
Güçel et al. 2012, Turkey	Low risk	Unclear risk	High risk	Low risk	Low risk	Unclear risk
Deng et al. 2014, China	Unclear risk	Unclear risk	High risk	Low risk	Low risk	Unclear risk
Hassan et al. 2014, Egypt	Unclear risk	Unclear risk	High risk	Low risk	Low risk	Unclear risk
He et al. 2014, China	Low risk	Unclear risk	High risk	Low risk	Low risk	Unclear risk
Wang et al. 2013, China	High risk	Unclear risk	High risk	Low risk	Low risk	Unclear risk
Sujung et al. 2014, South Korea	Low risk	Low risk	High risk	Low risk	Low risk	Unclear risk
Bu et al. 2007, China	Low risk	Unclear risk	High risk	Low risk	Low risk	Unclear risk
Shi et al. 2005, China	Low risk	Unclear risk	High risk	Low risk	Low risk	Unclear risk
Yang et al. 2010, China	High risk	Unclear risk	High risk	Low risk	Low risk	Unclear risk
Cabioglu et al. 2005, Turkey	Unclear risk	Unclear risk	High risk	Low risk	Low risk	Unclear risk
Cabioglu et al. 2006, Turkey	Unclear risk	Unclear risk	High risk	Low risk	Low risk	Unclear risk
Cabioglu et al. 2008, Turkey	Unclear risk	Unclear risk	High risk	Low risk	Low risk	Unclear risk
Darbandi et al. 2013, Iran	Low risk	Unclear risk	High risk	Low risk	Low risk	Unclear risk
Fogarty et al. 2015, Australia	Unclear risk	Low risk	High risk	Low risk	Low risk	Unclear risk

**Table 4:** Pairwise meta-analyses.

Comparison	Pairwise OR (95% CI)	Number of patients	Number of studies	Heterogeneity test	
				I^2^ (%)	p value
Body weight					
AR vs. LM	1.66(0.63 to 2.70)	496	10	55	0.02
AR vs. placebo	1.15(0.67 to 1.63)	833	14	65	0.0004
Combines therapies vs. PHA	2.44(-1.98 to 6.86)	80	1	-	-
Acupuncture vs. related therapies	0.25(0.00 to 0.49)	413	6	0	0.73
Combines therapies vs. acupuncture	1.56(0.07 to 3.05)	378	6	99	<0.00001
BMI					
AR vs. LM	1.17(0.09 to 2.26)	314	6	74	0.002
AR vs. placebo	0.57(0.40 to 0.74)	830	12	63	0.002
Combines therapies vs. PHA	0.48(-0.90 to 1.86)	80	1	-	-
Acupuncture vs. related therapies	0.13(-0.06 to 0.32)	325	5	0	0.8
Combines therapies vs. acupuncture	0.77(-0.37 to 1.92)	158	4	88	<0.00001

BMI: body mass index; LM: lifestyle modification; PHA: pharmacotherapy; AR: acupuncture and related therapies.

**Table 5:** Results of network meta-analyses.

Body weight									
**AAS**									
-1.11 (-4.01, 1.71)	**ACE**								
0.72 (-0.93, 2.37)	1.82 (-1.08, 4.84)	**AR+LM**							
-1.45 (-4.11, 1.28)	-0.36 (-1.82, 1.31)	-2.16 (-4.94, 0.61)	**Acupuncture**						
-2.40 (-5.16, 0.35)	-1.28 (-2.96, 0.39)	**-3.09 (-5.97, -0.33)**	-0.92 (-2.34, 0.30)	**Combined theories**					
-0.07 (-1.85, 1.74)	1.05 (-1.35, 3.48)	-0.77 (-2.61, 1.03)	1.38 (-0.77, 3.57)	**2.33 (0.17, 4.56)**	**EA**				
**1.80 (0.21, 3.41)**	**2.90 (0.16, 5.72)**	1.09 (-0.27, 2.39)	**3.26 (0.65, 5.88)**	**4.18 (1.62, 6.83)**	**1.85 (0.37, 3.37)**	**LM**			
0.06 (-5.43, 5.46)	1.11 (-3.79, 6.23)	-0.67 (-6.27, 4.70)	1.47 (-3.37, 6.35)	2.39 (-2.25, 7.16)	0.11 (-5.02, 5.13)	-1.76 (-7.18, 3.51)	**Pharmacotherapy**		
**1.26 (0.46, 2.06)**	2.37 (-0.43, 5.18)	0.54 (-0.93, 2.03)	**2.72 (0.06, 5.29)**	**3.65 (0.96, 6.34)**	1.33 (-0.36, 2.96)	-0.55 (-2.02, 0.90)	1.20 (-4.13, 6.63)	**Placebo**	
-3.47 (-8.46, 1.35)	-2.31 (-7.69, 2.72)	-4.18 (-9.22, 0.67)	-1.99 (-7.32, 2.93)	-1.04 (-6.34, 3.96)	-3.40 (-8.10, 1.06)	**-5.24 (-10.15, -0.55)**	-3.50 (-10.48, 3.39)	-4.72 (-9.77, 0.07)	**WA**

**BMI**									

**AAS**									
-0.08 (-1.54, 1.42)	**ACE**								
0.96 (-0.08, 2.00)	1.03 (-0.58, 2.66)	**AR+LM**							
-0.64 (-1.48, 0.35)	-0.54 (-1.88, 0.84)	**-1.59 (-2.71, -0.34)**	**Acupuncture**						
-0.81 (-1.77, 0.12)	-0.71 (-2.20, 0.70)	**-1.76 (-2.95, -0.62)**	-0.16 (-1.01, 0.43)	**Combined theories**					
0.04 (-0.68, 0.78)	0.12 (-1.20, 1.45)	-0.92 (-1.88, 0.08)	0.67 (-0.11, 1.34)	**0.84 (0.19, 1.58)**	**EA**				
**1.31 (0.36, 2.30)**	1.40 (-0.18, 2.96)	0.34 (-0.36, 1.15)	**1.94 (0.83, 3.00)**	**2.12 (1.07, 3.23)**	**1.28 (0.43, 2.11)**	**LM**			
-0.31 (-2.14, 1.55)	-0.21 (-2.34, 1.87)	-1.26 (-3.21, 0.79)	0.33 (-1.46, 2.01)	0.51 (-1.06, 2.09)	-0.35 (-2.10, 1.39)	-1.62 (-3.54, 0.31)	**Pharmacotherapy**		
**0.63 (0.25, 1.11)**	0.72 (-0.71, 2.15)	-0.32 (-1.28, 0.69)	**1.28 (0.43, 2.05)**	**1.44 (0.64, 2.38)**	**0.60 (0.03, 1.22)**	-0.66 (-1.58, 0.26)	0.95 (-0.85, 2.78)	**Placebo**	

BMI: body mass index; LM: lifestyle modification; AAS: auricular acupoint stimulation; EA: electroacupuncture; ACE: acupoint catgut embedding; WA: warming acupuncture; AR: acupuncture and related therapies; combined therapies: combination of acupuncture and related therapies.

## Data Availability

All data used to support the findings of this study are included within the supplementary information files.

## Supplementary Materials

Supplementary Materials Appendix 1: the PRISMA-NMA checklist treatment groups for included studies in a network meta-analysis. Appendix 2: search strategies for RCTs on acupuncture for obesity. Appendix 3: summaries of mean, standard difference, and sample size between treatment groups for included studies in a network meta-analysis. Appendix 4: inconsistency test.
